# Estimating the Occurrence of Wind-Driven Coastal Upwelling Associated with “Aoshio” on the Northeast Shore of Tokyo Bay, Japan: An Analytical Model

**DOI:** 10.1155/2014/769823

**Published:** 2014-01-20

**Authors:** Zhongfan Zhu, Jingshan Yu

**Affiliations:** College of Water Sciences, Beijing Normal University, Xinjiekouwai Street 19, Beijing 100875, China

## Abstract

“Aoshio” in Tokyo Bay is a hydroenvironmental phenomenon in which seawater appears milky blue due to reflection of sunshine off surface water which contains lots of sulfur particles. Its appearance is due to coastal upwelling of bottom oxygen-depleted water, which causes many deaths of shellfish and other aquatic animals around the bay. In this study, we derived some analytical solutions in the context of a two-layered fluid and used them to make a simple analytical model to estimate the occurrence of “Aoshio” phenomenon on the northeast shore of Tokyo Bay. Comparison with observation data suggested that this model was valid to a certain degree.

## 1. Introduction

At the head of Tokyo Bay, continual blowing of a northeasterly wind often causes coastal upwelling of oxygen-depleted bottom water, which leads to many deaths of shellfish and other aquatic animals around the bay. During this process, a large amount of hydrogen sulfide originally contained in the bottom water is oxidized to colloidal sulfur particles when it touches the oxygen in the atmosphere near the surface. When sunshine reflects off surface water in which these sulfur particles suspend, the color of seawater presents as milky blue, and this phenomenon is termed “Aoshio” (in Japanese, “Ao” means blue, and “Shio” means tide).

Many researches concerning “Aoshio” phenomenon [1–5] have been done. Based on these research results, we attempt to carry out the quantitative calculation of this phenomenon, especially wind conditions under which it can appear at the head of Tokyo Bay. In the preliminary study, starting from governing equations of a two-layered fluid, we derived some analytical solutions and used them to make a simple analytical model to estimate whether “Aoshio” phenomenon can happen on the northeast shore of the bay. Compared with numerical simulation, the analytical method adopted here can be more helpful to roughly evaluate wind conditions under which “Aoshio” can occur at Tokyo Bay and obtain a qualitative understanding of the factors that can influence the occurrence of “Aoshio”, although it is based on several simplifications and assumptions.

The layout of the paper is as follows.  Section 2 briefly introduces engineering background and derivations of analytical solutions.  Section 3 presents comparison of the analytical model with observational data.  Section 4 contains concluding remark, and additionally the appendix presents the mathematical derivations of the mentioned analytical solutions.

## 2. Engineering Background and Derivations of Analytical Solutions

### 2.1. Engineering Background

**Figure 1:**
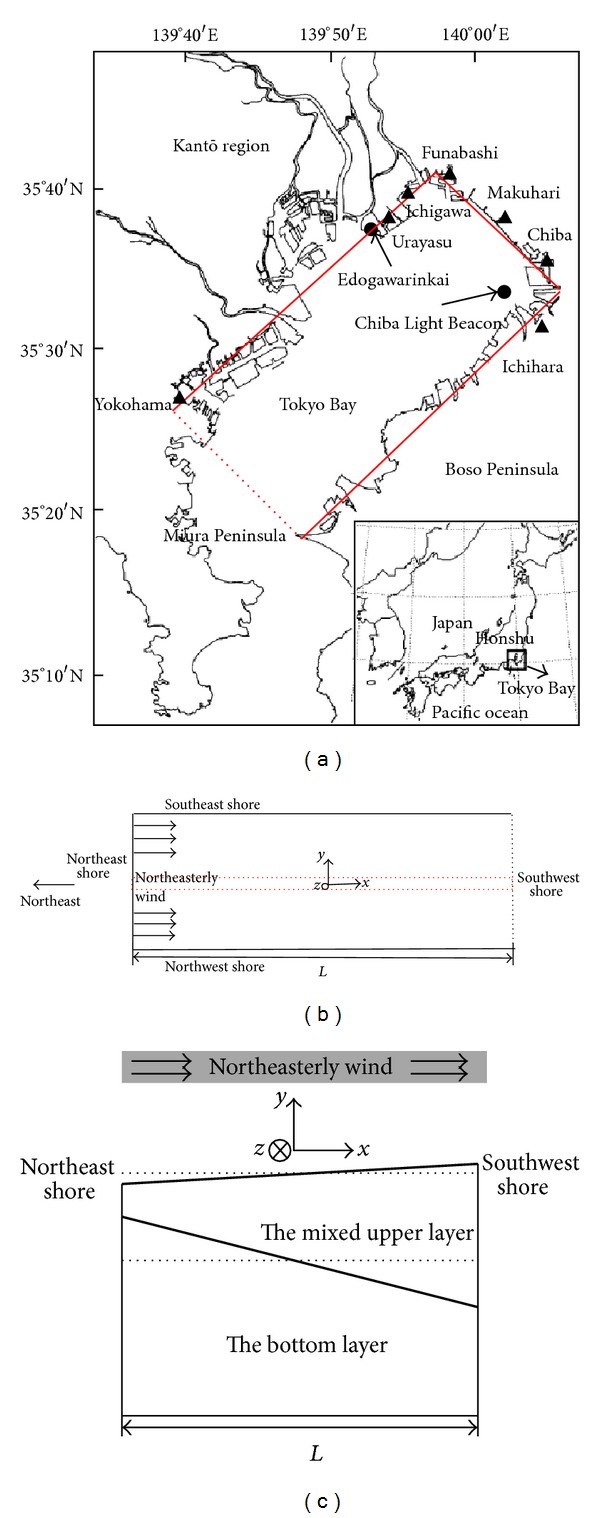
(a) Map of Tokyo Bay including the chosen research domain; (b) dimensions of the domain; (c) schematic diagram of coastal upwelling on the northeast shore, caused by the blowing of a northeasterly wind [6].

Facing the Pacific Ocean, Tokyo bay (Figure 1(a)) is located in the southeastern part of Honshu island of Japan. Its main axis length, mean width, and average depth are approximately 60 km, 20 km, and 15 m, respectively. Due to continual blowing of a northeasterly wind, coastal upwelling associated with “Aoshio” phenomenon can be observed along the coastline from off Funabashi to off Chiba.

Although actual coastline is complicated, we still simply consider the research domain to be a rectangular box with the length of 50 km, the width of 20 km and the depth of 15 m respectively, as shown in Figure 1(a) by three solid lines and a dashed line indicating the imaginary southwest shore [6]. Dimensions of this chosen domain are illustrated in Figure 1(b), where *L* denotes the length, and the Cartesian coordinate system is also defined (the origin is in the center of the domain, the *x* axis follows the northeasterly wind with the *y* axis perpendicular to it, and the *z* axis is positive in the upward direction with respect to the still surface level).

Using a two-layered fluid model which is composed of a well-mixed upper layer and an oxygen-depleted bottom layer with different densities (these two layers are separated by a density interface), Zhu and Isobe [6] analyzed the full process of the occurrence of coastal upwelling associated with “Aoshio.” When a northeasterly wind suddenly starts to blow uniformly across the static fluid in the domain, coastal upwelling will occur on the northeast shore, as long as the wind conditions are enough. With the continual presence of the wind, the effect due to the Coriolis force will be predominant, and coastal upwelling may appear on the southeast shore. The time scale after which the Coriolis force becomes predominant is empirically considered, and it is practicable to consider this time scale to be two days, based on the numerical experiment of coastal upwelling in Tokyo Bay carried out by Matsuyama et al. [1] and the statistics of “Aoshio” on the southeast shore of the bay. Wind conditions for the occurrence of upwelling on the southeast shore of the bay have been discussed by Zhu and Isobe [6] and we only focus on upwelling on the northeast shore in this study.

For the analysis of upwelling on the northeast shore, we choose a water parcel as indicated by dashed lines in Figure 1(b) and analyze the motion of this water parcel using the mentioned two-layered fluid model as schematically shown in Figure 1(c). Sudden blowing of a northeasterly wind accelerates this water parcel in the downwind direction, causing the surface level to rise on the southwest shore and fall on the northeast shore. The pressure gradient due to inequality of the surface level at these two shores will cause the oxygen-depleted water beneath the interface to move toward the northeast shore until it disappears, finally leading to the tilt of the interface toward the northeast shore. If some wind conditions are satisfied, the interface will reach the surface level. At this point, the oxygen-depleted bottom water can touch the oxygen in the atmosphere, and “Aoshio” phenomenon will appear on the northeast shore of the domain.

### 2.2. Derivations of Analytical Solutions

Starting from governing equations of the mentioned two-layered model, we can attempt to derive the mathematical expression of wind conditions under which upwelling occurs on the northeast shore. Solving directly these governing equations seems difficult, and in this study we adopt the following method as presented in Csanady [7]: governing equations can be transformed into an internal mode and a surface mode; for each mode, governing equations can be solved, and the solution of any mentioned parameter should be a simple sum of its solution in the internal mode and that in the surface mode. All of discussions regarding how these modes are defined and derived in detail can be found in Csanady [7]. Compared to those already introduced, we add both terms of interfacial friction and bottom friction which normally should not be neglected.

Governing equations of the internal mode are

(1) ∂Ui∂t=gεhh′h+h′∂ζi′∂x+h′h+h′(τwρ0−τIiρ)−hh+h′(τIiρ′−τBiρ′),

(2) ∂Ui∂x=∂ζi′∂t,

and this mode is defined with some relations

(3) Ui+Ui′=0,  ζi=−εh′h+h′ζi′.

In these equations, (*U*_*i*_, *U*_*i*_′) are vertically integrated horizontal velocity in the *x* direction for the upper layer and the lower one in the internal mode, respectively, *h*, *h*′ are thicknesses of the upper layer and the lower one, respectively, *ρ*, *ρ*′ are densities of the upper layer and the lower one, respectively, *ρ*_0_ is reference density of water, *ζ*_*i*_, *ζ*_*i*_′ are surface displacement and interfacial displacement in the internal mode, respectively, *g* and *ε* are gravitational acceleration and density contrast between two layers (*ε* = (*ρ*′ − *ρ*)/*ρ*′), *τ*_*w*_ is stress of the northeasterly wind, (*τ*_*Ii*_, *τ*_*Bi*_) are interfacial friction and bottom friction in the *x* direction in the internal mode, respectively, and *t* is time.

Governing equations of the surface mode are

(4) ∂∂t(Us+Us′)=−g(h+h′)∂ζs∂x+(τwρ0−τIsρ)+(τIsρ′−τBsρ′),∂∂x(Us+Us′)=−∂ζs∂t,

and there exists some relations in this mode

(5) Ush=Us′h′,  ζs′=h′h+h′ζs.

In these equations, all of the quantities with the subscript “*s*” have same meanings as introduced above, but denote the parameters in the surface mode.

Interfacial friction term is assumed to be expressed in terms of the velocity difference between the upper layer and the lower one here as

(6) τIi≈ρCI(Uih−Ui′h′)=ρ′CI′(Uih−Ui′h′),τIs≈ρCI(Ush−Us′h′)=ρ′CI′(Ush−Us′h′),

where *C*_*I*_, *C*_*I*_′ are interfacial friction coefficients of the upper layer and the lower one, respectively; similarly, bottom friction term is assumed to be expressed in terms of the velocity of the lower layer as

(7) τBi≈ρ′CBUi′h′,  τBs≈ρ′CBUs′h′,

where *C*_*B*_ is bottom friction coefficient.

Substituting (6) and (7) into (1) and using (3) can yield

(8) ∂Ui∂t+k1Ui=gεhh′h+h′∂ζi′∂x+h′h+h′τwρ0,

where a new parameter *k*_1_ is defined as *k*_1_ = *C*_*I*_/*h* + *C*_*I*_′/*h*′ + *C*_*B*_ *h*/[*h*′(*h* + *h*′)], and this parameter expresses the influences of interfacial friction and bottom friction. By virtue of the Laplace-transform method, analytical solutions to (8) and (2) subject to boundary conditions that *U*_*i*_(*x*, *t*) = 0 at *x* = ±*L*/2 can be obtained as following: (a brief derivation can be found in the appendix)

(9) ζi′(x,t)=−1gεhτwρ0x+1gεhτwρ04Lπ2e−(k1/2)t ×∑n=1+∞(−1)n−1sin((2n−1)πx/L)(2n−1)2    ×((k1/2)sinhσnt+σncoshσnt)σn,

(10) Ui(x,t)=4h′(h+h′)τwρ0e−(k1/2)t ×∑n=1+∞(−1)n−1cos((2n−1)πx/L)(2n−1)πsinhσntσn,

where a parameter *σ*_*n*_ is defined as $\sigma_{n}=\sqrt{k_{1}^{2}-4g\varepsilon hh^{\prime }/\left(h+h^{\prime }\right){\left(2n-1\right)}^{2}\pi^{2}/L^{2}}/2,\;\;n=1,2,3,\ldots$, and this parameter can be regarded as a parameter related to the oscillation of surface displacement or interfacial displacement in the internal mode. With (9) and (10), expressions of other parameters in the internal mode can be obtained according to (3). The right side of (9) generally consists of two terms: the first term is independent of time *t*, and the other term is a complicated function of time. It can be inferred that this complicated function approaches zero when the time is infinitely long (*t* → *∞*), implying that the first term is a steady-state solution and the other one expresses the manner in which this steady state is reached. All that remain are *U*_*i*_ → 0 in (10) and *U*_*i*_′ → 0 inferred from both of (10) and (3) when time is infinitely long (*t* → *∞*), meaning that there is a vertical circulation in each of the upper layer and the lower one at the steady state.

Substituting (6) and (7) into (4) and using (5) one can have

(11) ∂Us∂t+CBh+h′Us=−gh∂ζs∂x+hh+h′τwρ0,∂Us∂x=−hh+h′∂ζs∂t.

Similarly, solving these equations subject to boundary conditions that *U*_*s*_(*x*, *t*) = 0 at *x* = ±*L*/2 by using the Laplace-transform method one can get

(12) ζs(x,t)=1g(h+h′)τwρ0x−1g(h+h′)τwρ04Lπ2e−(CB/2(h+h′))t×∑n=1+∞(−1)n−1sin((2n−1)πx/L)(2n−1)2  ×((CB/2(h+h′))sinhσn¯t+σn¯coshσn¯t)σn¯,

(13) Us(x,t)=4h(h+h′)τwρ0e−(CB/2(h+h′))t×∑n=1+∞(−1)n−1cos((2n−1)πx/L)(2n−1)πsinhσn¯tσn¯,

where a new parameter $\bar{\sigma_{n}}$ is defined as $\bar{\sigma_{n}}=\sqrt{{\left[C_{B}/\left(h+h^{\prime }\right)\right]}^{2}-4g\left(h+h^{\prime }\right){\left(2n-1\right)}^{2}\pi^{2}/L^{2}}/2$, *n* = 1,2, 3,…, and this parameter can be regarded as a parameter related to the oscillation of surface displacement or interfacial displacement in the surface mode. With (12) and (13), expressions of other parameters in the surface mode can be obtained according to (5). Similar to (9), the right side of (12) is also composed of a steady-state solution and the term expressing the manner in which this steady state is approached. The vanishing *U*_*s*_(*x*, *t*) from (13) and *U*_*s*_′(*x*, *t*) which can be inferred from both (13) and (5) when time is infinitely long (*t* → *∞*) imply the existence of a vertical circulation in each of the upper and lower layers at the steady state.

Total expression of surface displacement *ζ*_total_(*x*, *t*) is the sum of the right-hand side of (9) multiplied by the factor −*εh*′/(*h* + *h*′) and the right-hand side of (12), and total expression of interfacial displacement *ζ*_total_′(*x*, *t*) is the sum of the right-hand side of (9) and the right-hand side of (12) multiplied by the factor *h*′/(*h* + *h*′).

When upwelling only occurs on the northeast shore at a time *t* = *T* (*T* is duration of the northeasterly wind), the sum of absolute values of surface displacement and interfacial displacement should simply be equal to the thickness of the upper layer (|*ζ*_total_(−*L*/2, *T*)| + *ζ*_total_′(−*L*/2, *T*) = *h*), and a wind stress can be gotten. Consider

(14) τwρ0=h×{4Lghπ2(1ε+h′h+h′)  ×[π28−e−(k1/2)T   ×∑n=1+∞1(2n−1)2    ×((k1/2)sinhσnT+σncoshσnT)σn]   +4Lgπ2h(h+h′)2   ×[π28−e−(CB/2(h+h′))T    ×∑n=1+∞1(2n−1)2    ×((CB/2(h+h′))sinhσn¯T+σn¯coshσn¯T)σn¯]}−1≈h(4Lghπ2(1ε+h′h+h′)   ×[π28−e−(k1/2)T   ×∑n=1+∞1(2n−1)2    ×((k1/2)sinhσnT+σncoshσnT)σn])−1≈gεh2π2 ×(4L[π28−e−(k1/2)T    ×∑n=1+∞1(2n−1)2   ×((k1/2)sinhσnT+σncoshσnT)σn])−1.

In this expression, the reason why the first approximate equality exists is that the term 4*L*/(*ghπ* ^2^)[1/*ε* + *h*′/(*h* + *h*′)] is much larger than the term 4*Lh*/[*gπ* ^2^(*h*+*h*′)^2^] in the denominator term, implying that the contribution of the surface mode to surface displacement or interfacial displacement can be negligible compared with the internal mode. The reason why the second approximate equality exists is that the term 1/*ε* is much larger than the term *h*′/(*h* + *h*′), meaning that surface displacement can be neglected compared with interfacial displacement. Furthermore, it can be inferred that, if wind duration *T* is fixed, increasing *τ*_*w*_/*ρ*_0_ yields larger values for |*ζ*_total_(−*L*/2, *T*)| and *ζ*_total_′(−*L*/2, *T*). This means that (14) actually represents the minimum wind stress for the occurrence of coastal upwelling associated with “Aoshio” on the northeast shore of the domain. In addition, (14) can be expressed to be *g*′*h* ^2^/(*u*_∗_^2^ *L*) ≈ 1/2 − 4/*π* ^2^ × *e* ^−*k*_1_*T*/2^∑_*n*=1_^+*∞*^1/(2*n* − 1)^2^(*k*_1_sinh*σ*_*n*_ *T*/2 + *σ*_*n*_cosh*σ*_*n*_ *T*)/*σ*_*n*_, where *g*′ is the reduced gravitational acceleration (*g*′ = *gε*) and *u*_∗_ is the shear velocity of the surface-wind stress at the fluid side ($u_{*}=\sqrt{\tau_{w}/\rho_{0}}$). The term *g*′*h* ^2^/(*u*_∗_^2^ *L*) has been termed as the Wedderburn number (e.g., Shintani et al. [8]), and it consists of the Richardson number and the aspect ratio of the domain *h*/*L*.

## 3. Comparison with Observation Data 

### 3.1. Presentation of the Model

The term of the wind stress in (14) can be expressed to be a quadratic function of wind speed [1]:

(15) τw=ρaγa2w2,

where *ρ*_*a*_ is the air density, *γ*_*a*_^2^ is the surface drag coefficient, and *w* is average wind speed which is measured 10 m above the still surface.

In order to present (14), we assign some empirical values to unknown parameters in this equation, as presented in Table 1 also including known parameters. In this table, a simple estimate for *C*_*I*_, *C*_*B*_ shows that they should be on the order of 10^−6^–10^−5^, so we have chosen the empirical value 0.50 × 10^−5^. Values for densities and thicknesses of the upper layer and the lower one are only adopted as an example, and they originate from the numerical experiment of Matsuyamaet al. [1], where they are representative of the typical stratification of Tokyo Bay in summer.

**Figure 2:**
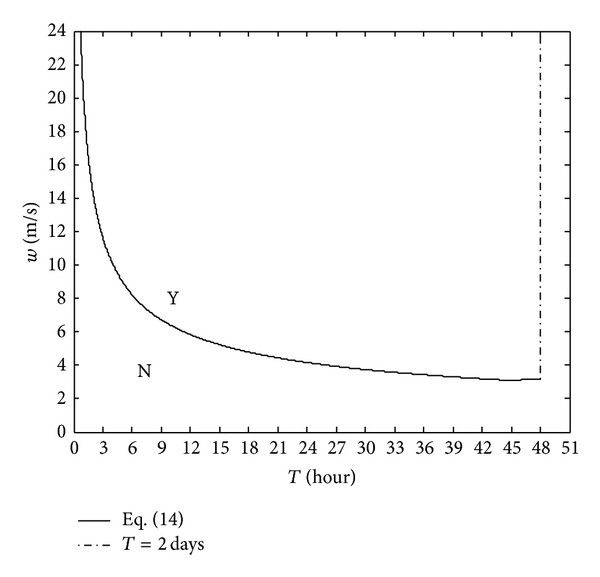
Presentation of the analytical model using typical parameter values in in Table 1.

Equation (14) is presented in Figure 2 and in this figure the combination of (14) and the line *T* = 2 days divides the entire region into two different regions. Region Y is the region in which upwelling can occur on the northeast shore of Tokyo bay, and region N is the region without upwelling on this shore. If the wind speed and the wind duration are known, it is possible to estimate whether upwelling associated with “Aoshio” can appear on the northeast shore of the bay using this figure.

### 3.2. Treatment of Observation Data

**Figure 3:**
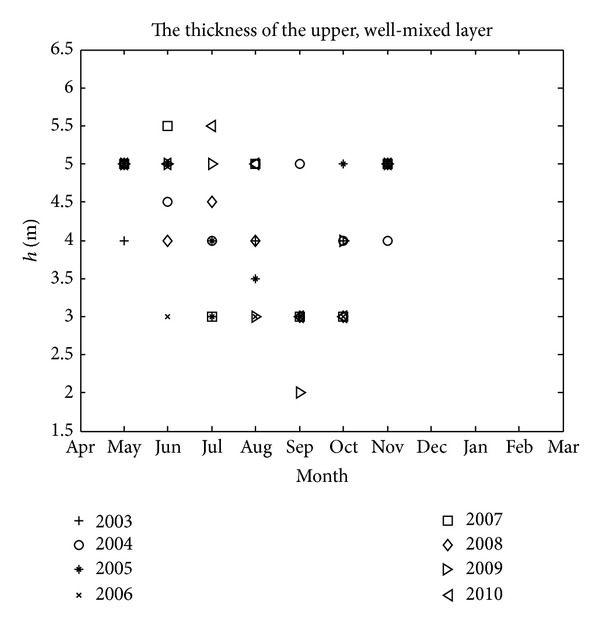
Monthly-mean thickness value of the upper well-mixed layer from 2003 to 2010.

Observation data of “Aoshio” in Tokyo Bay from 1978 to 2010 are available, and they originate from three different sources [6]. Among these, we selected data sets of “Aoshio” only observed on the northeast shore. We adopted data sets of wind measured at Edogawarinkai station which is indicated in Figure 1(a), and further calculated average speed and total duration of the northeasterly-oriented wind that contributes to the occurrence of “Aoshio” [6]. For all of the real cases in which “Aoshio” appeared on the northeast shore, all of the parameter values can be treated as constants as presented in Table 1, except for the thickness of the upper well-mixed layer *h* and the density contrast *ε*. This is because these two parameters are closely related to the degree of stratification, and they should be different from one case to another. In order to calculate them for each case, we used real-time data measured at the Chiba Light Beacon (CLB) also indicated in Figure 1(a) [6]. The thickness of the well-mixed upper layer can be estimated from vertical distribution contours of temperature, salinity, and dissolved oxygen, and the densities of the upper and lower layers can be calculated based on the temperature and the salinity. However, these data sets provided at CLB only range from 2003 to 2010. For those real cases from 1978 to 2002 that lack real-time data, we adopted the following strategy as presented in Zhu and Isobe [6]: we focused on the seasonal variation of stratification, and considered the algebraically averaged thickness values and density values for each month from 2003 to 2010 to be representative of real cases observed from 1978 to 2002. Calculated monthly-mean densities and algebraically averaged densities of the upper layer and the lower one for each month have been provided in Zhu and Isobe [6], and monthly-mean thickness and algebraically averaged thickness of the upper well-mixed layer are shown in Figure 3 and Table 2, respectively. In addition, it needs to be stated that this study did not use the spatial averaged wind data and stratification data in Tokyo Bay because of a lack of long-term real-time data measured at different locations in the bay beyond CLB.

### 3.3. Comparison Result

**Figure 4:**
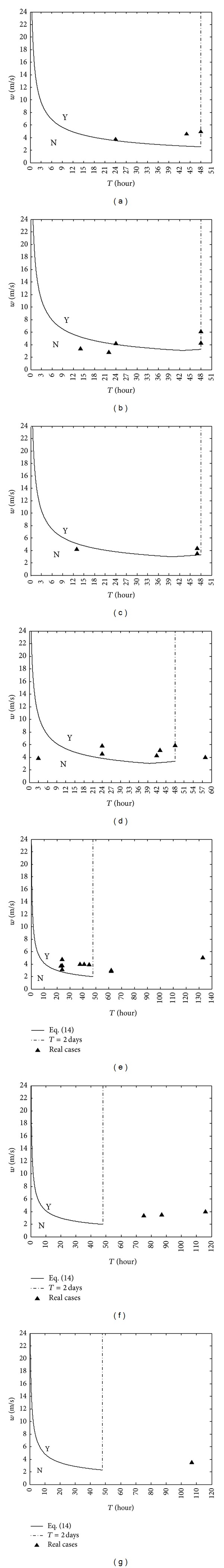
Comparison of the model with real cases of Aoshio observed from 1978 to 2002 in a particular month. (a) May, (b) June, (c) July, (d) August, (e) September, (f) October, and (g) November.

Table 3 summarizes the comparison result of the model with real cases of “Aoshio” observed on the northeast shore of Tokyo Bay from 2003 to 2010. The first column shows occurrence date and occurrence area of “Aoshio” (In “Occurrence area” column, the expression “A-B” denotes the area which ranges from A to B hereafter). The second column presents the calculated average wind speed in the northeast-southwest direction and the wind duration. The third column show the degree of stratification using measurement data from CLB. Using these values, the fourth column presents the calculated model. Conclusions about whether each of real cases is in accord with the model are shown in the fifth column, suggesting that one of three real cases agrees with the proposed model. Figures 4(a)–4(g) shows the comparison result of the model with real cases from 1978 to 2002 using the representative values of each month. From these figures, it can be found that almost sixty-three percent of real cases are consistent with the model. As an addition, Table 4 presents occurrence dates and occurrence areas of these real cases, as well as wind conditions. Among all of real cases which did not agree with the model, it can be found that the duration of the northeasterly-oriented wind exceeds two days in nine real cases (as presented in the last row of Table 3 and Figures 4(d)–4(g)), for which the effect due to the Coriolis force cannot be simply negligible. We compared them with the criteria proposed by Zhu and Isobe [6] for the occurrence of “Aoshio” on the southeast shore of the bay as presented in the last column in Table 3 and the third column of Table 4 and further found that “Aoshio” can happen on the southeast shore in all of these cases. The appearance of the phenomenon that “Aoshio” area was not on the southeast shore but on the northeast shore may be due to the anticlockwise movement of “Aoshio” area from the southeast shore to the northeast shore under the influence of the propagation of internal Kelvin waves after the cessation of the northeasterly wind, as clearly presented in the numerical simulation of Matsuyama et al. [1] and further pointed out in Ueno et al. [4] and Nakatsujiet al. [5]. More analyses about the role that internal Kelvin waves play in the movement of “Aoshio” area were not mentioned in this study and will be performed in future research. The related discussions for those real cases which neither satisfy the criteria for “Aoshio” on the southeast shore nor agree with the analytical model (they are cases of that “Aoshio” occurred in June 13, 1979, August 24, 1984, June 1 and July 2, 1992, and May 16, 2005) were not carried out in this preliminary study because of a lack of the detailed observation data regarding these cases (especially the detailed descriptions of the movement of the oxygen-depleted bottom water in these cases). In conclusion, considering that several simplifications and assumptions were used, it can be suggested that the analytical model proposed here is valid to a certain degree.

## 4. Concluding Remarks

In this study, we have derived some analytical solutions in the context of a two-layered fluid and have used them to make a simple analytical model to estimate the occurrence of “Aoshio” phenomenon on the northeast shore of Tokyo Bay. Comparison with observational data suggested that this model was valid to a certain degree.

## Figures and Tables

**Table 1:** Typical parameter values for Tokyo Bay.

The parameter	Value
Thickness of the upper layer *h*	5 m
Thickness of the lower layer *h*′	10 m
Density of the upper layer ρ	1020 kg/m^3^
Density of the lower layer ρ′	1023 kg/m^3^
Density contrast *ε*	2.93 × 10^−3^
Length of the bay *L*	50 km
Reference density ρ_0_	1000 kg/m^3^
Air density ρ_*a*_	1.23 kg/m^3^
Surface drag coefficient γ_*a*_^2^	1.30 × 10^−3^
Interfacial friction coefficient of the upper layer *C*_*I*_	0.50 × 10^−5^ m/s
Interfacial friction coefficient of the lower layer *C*_*I*_′	0.50 × 10^−5^ m/s
Bottom friction coefficient *C*_*B*_	0.50 × 10^−5^ m/s
The parameter expressing the influences of interfacial friction and bottom friction *k*_1_	1.67 × 10^−6^ s^−1^

**Table 2:** Algebraically averaged thickness value of the upper well-mixed layer for each month, calculated based on real-time data taken from 2003 to 2010.

	May	Jun.	Jul.	Aug.	Sep.	Oct.	Nov.
The thickness of the upper, well-mixed layer (m)	4.88	4.63	4.00	4.06	3.13	3.63	4.88
The thickness of the lower layer (m)	10.13	10.38	11.00	10.94	11.88	11.38	10.13

**Table 3:** Comparison of the model with real cases of Aoshio observed on the northeast shore of Tokyo Bay from 2003 to 2010 (based on measured data from Chiba Light Beacon).

Upwelling associated with Aoshio on the northeast shore of Tokyo Bay			Northeasterly wind	Degree of stratification						Calculated model proposed in this study	Does the real case satisfy the calculated model?	Does the real case satisfy the criteria for Aoshio on the southeast shore proposed by Zhu and Isobe (2012) [6]?
Occurrence date		Occurrence area	Average wind speed/wind duration (m/s)/(h)	Surface temperature /surface salinity (°C)/(psu)	Bottom temperature /bottom salinity (°C)/(psu)	Density of the upper layer (kg/m^3^)	Density of the lower layer (kg/m^3^)	The thickness of the upper layer (m)	The thickness of the lower layer (m)		
2005	May 16-May 17	Urayasu-Ichigawa-Funabashi	2.99/18	15.6/32.2	15.6/33.4	1023.71	1024.63	5.00	10.00	*T* ≤ 2 days, *w* ^2^ ≥ 12.7311 m^2^/s^2^	No	—
2007	Oct. 1-Oct. 2	Funabashi	3.76/48	21.6/29	21.6/32.8	1019.80	1022.69	5.00	10.00	*T* ≤ 2 days, *w* ^2^ ≥ 9.7013 m^2^/s^2^	Yes	—
2008	Nov. 13-Nov. 14	Funabashi-Ichigawa	3.38/95	18/32.2	19.2/33.00	1023.16	1023.47	5.00	10.00	*T* ≤ 2 days, —	No	Yes

**Table 4:** Occurrence dates, occurrence areas, and wind conditions of real cases of Aoshio observed on the northeast shore of Tokyo Bay from 1978 to 2002.

Upwelling associated with Aoshio on the northeast shore of Tokyo Bay			Northeasterly wind	Does the real case satisfy the criteria for Aoshio on the southeast shore proposed by Zhu and Isobe (2012) [6]?
Occurrence date		Occurrence area	Average Wind speed/wind duration (m/s)/(h)
1978	May 31	Urayasu-Funabashi	3.73/24	—
1979	Jun. 13	Funabashi	3.36/14	—
	Jul. 16-Jul. 17	Makuhari	4.30/47	—
	Aug. 14–Aug. 16	Makuhari	4.25/42	—
1980	Aug. 2–Aug. 5	Funabashi-Makuhari	5.80/24	—
	Sep. 19	Funabashi	3.96/41	—
1982	May. 21–May. 23	Ichigawa	4.55/44	—
	Jul. 27–Jul. 29	Ichigawa-Makuhari	3.50/47	—
	Sep. 6	Funabashi	5.03/133	Yes
1984	Aug. 24–Aug. 26	Funabashi-Makuhari	3.82/3	—
	Sep. 9	Funabashi	3.12/24	—
1985	Jun. 15–Jun. 18	Funabashi	6.07/48	—
1986	Aug. 4	Urayasu-Ichigawa	3.94/58	Yes
1988	May 24	Funabashi	4.94/48	—
	Sep. 3–Sep. 8	Funabashi	3.75/24	—
1989	Jun. 20	Funabashi	4.28/48	—
	Aug. 26-Aug. 27	Funabashi	4.50/24	—
	Sep. 22–Sep. 24	Funabashi-Makuhari	3.92/45	—
	Oct. 30	Funabashi-Makuhari	3.35/75	Yes
1990	Jun. 28–Jul. 2	Funabashi-Makuhari	4.18/24	—
	Aug. 6	Ichigawa-Funabashi-Makuhari	5.89/48	—
	Sep. 7-Sep. 8	Urayasu-Funabashi	4.72/24	—
	Sep. 27–Sep. 29	Funabashi-Makuhari	3.99/38	—
1992	Jun. 1	Funabashi-Makuhari	2.80/22	—
	Jul. 2	Funabashi	4.18/13	—
	Aug. 3–Aug. 6	Ichigawa-Funabashi	5.12/43	—
1994	Nov. 4–Nov. 8	Makuhari-Funabashi	3.45/107	Yes
1996	Sep. 10–Sep. 12	Funabashi-Makuhari	3.71/23	—
1999	Sep. 30	Funabashi	2.97/62	Yes
	Oct. 18–Oct. 20	Funabashi	3.46/87	Yes
2000	Sep. 27–Sep. 29	Funabashi	2.85/62	Yes
2001	Apr. 23-Apr. 24	Funabashi	4.39/24	—
	Oct. 9-Oct. 10	Makuhari and Funabashi and Ichigawa	3.99/116	Yes

## Appendix

Taking the derivative of (2) with respect to *t* and the derivative of (8) with respect to *x*, respectively, and combing them can yield

(A.1) ∂2ζi′∂t2+k1∂ζi′∂t=gεhh′h+h′∂2ζi′∂x2.

Introducing the Laplace transform of a arbitrary function *φ*(*t*) as *Q*(*φ*) = ∫_0_^*∞*^ *e* ^−*st*^ *φ*(*t*)*dt* (*s* is a parameter in the transformation), (A.1) can be transformed to be

(A.2) gεhh′h+h′d2Q(ζi′)dx2−(s2+k1s)Q(ζi′)=0.

The analytical solution of this equation, complying with the boundary conditions, is

(A.3) Q(ζi′)=−1gεhτwρ01s×1(s2+k1s)(h+h′)/gεhh′×sinh[((s2+k1s)(h+h′)/gεhh′)x]cosh[((s2+k1s)(h+h′)/gεhh′)(L/2)].

To invert this Laplace transform, we define such a function *f*(*x*, *t*) so that

(A.4) Q(f(x,t))=1(s2+k1s)(h+h′)/gεhh′×sinh[((s2+k1s)(h+h′)/gεhh′)x]cosh[((s2+k1s)(h+h′)/gεhh′)(L/2)].

This function is analytic everywhere except at a series of poles, *s* = 0, *s* = −*k*_1_, and *s* = −*k*_1_/2 ± *σ*_*n*_, where

(A.5) σn=k12−4gεhh′/(h+h′)(2n−1)2π2/L22,n=1,2,3,….

The residues at *s* = 0 and *s* = −*k*_1_ are zero.

The residues at *s* = −*k*_1_/2 + *σ*_*n*_ and *s* = −*k*_1_/2 − *σ*_*n*_ may be combined to be

(A.6) Residues  at  s=−k12±σn=2gεhh′σnL(h+h′)sin((2n−1)πx/L)sin(n−1/2)π ×[e(−k1/2+σn)t−e(−k1/2−σn)t],

which gives the expression of the stated function *f*(*x*, *t*) by using the residue theorem. Using the convolution theorem, the function *ζ*_*i*_′(*x*, *t*) in (A.3) can be gained (it can be found that *Q*(1) = 1/*s* in this equation). The function *U*_*i*_(*x*, *t*) can also be obtained by combining (2).
